# Long‐Term Self‐Reported Symptoms Among Adults After COVID‐19 Infection in the West Bank: A Cross‐Sectional Analysis

**DOI:** 10.1155/ghe3/2867843

**Published:** 2025-12-11

**Authors:** Tareq Jarrar, Noor Halaseh, Duha Doudin, Peter Bael, Atheer Shaheen, Eyad Jobeh, Ahmad Abu Sada, Ahmad Abu Awwad, Bayan Alqtishat, Hussein Hallak

**Affiliations:** ^1^ Medical Research Club, Faculty of Medicine, Al-Quds University, Jerusalem, State of Palestine, alquds.edu; ^2^ Department of Pharmacology, Faculty of Medicine, Al-Quds University, Jerusalem, State of Palestine, alquds.edu

**Keywords:** COVID-19, COVID-19 symptoms, long COVID, pandemic

## Abstract

**Introduction:**

With growing recognition of the prolonged effects of COVID‐19, there is an urgent need to understand its extended clinical and public health implications across diverse settings. Long‐term consequences following SARS‐CoV‐2 infection remain insufficiently studied in Middle Eastern populations. This study aimed to assess the prevalence of persistent COVID‐19 symptoms among Palestinian adults and to evaluate their associations with hospitalization and recovery duration.

**Methods:**

This cross‐sectional study was conducted on a randomized sample of 407 adult COVID‐19 patients confirmed by the Palestinian Ministry of Health between November 25 and December 15, 2020. We used a standardized Arabic questionnaire to cover demographics, medical history, symptoms, complications, and physical activity. Data were gathered by phone interviews in October 2021. All data came from self‐reports. With significance defined at *p* < 0.05, associations between the symptom duration, hospitalization, and recovery time were examined using descriptive statistics and chi‐square tests.

**Results:**

The study population had a mean age of 40 years; 54% were female, and 70.3% had no previous medical history. Common complaints were fatigue (64.9%), anosmia (61.9%), joint pain (52.6%), and headache (51.8%). Hospitalization was necessary in 7.6% of patients, while 5.9% required oxygen or intubation. Most patients (92.6%) recovered in 4 months. The persistence of chest pain (*χ* ^2^, 16.225), shortness of breath (*χ* ^2^, 13.257), and lethargy (*χ* ^2^, 8.194) was significantly associated with hospitalization (*p* < 0.001). The persistence of the previously mentioned symptoms was significantly associated with the duration of recovery.

**Conclusion:**

This study offers valuable insights into the long‐term symptoms experienced by individuals recovering from COVID‐19 in the West Bank. The findings carry implications for clinicians, public health authorities, and affected individuals, highlighting the importance of integrated care strategies and sustained support throughout the postacute phase of the disease.

## 1. Introduction

December 2019 had become synonymous with the global health pandemic of modern times, COVID‐19. It is traced to the SARS‐CoV‐2 virus, a coronavirus that can cause various illnesses. While some people may only experience mild symptoms similar to those of a common cold, others may develop severe conditions with significant morbidity and mortality [1].

The World Health Organization (WHO) estimated that over 777.4 million people were infected and over seven million have lost their lives [2]. The virus was initially reported in Wuhan, Hubei, China, with respiratory symptoms such as fever, dyspnea, and dry cough being the most common complaints. It also affected other extrapulmonary systems, leading to renal failure, heart disease, and neurological disorders [3]. However, the virus has since mutated, with new strains emerging and subsequently diversifying both symptoms and severity [4].

According to the WHO, post‐COVID‐19 condition, often referred to as long COVID, is defined as the continuation or development of new symptoms 3 months after the initial SARS‐CoV‐2 infection, with these symptoms persisting for at least 2 months and not explained by alternative diagnoses [5]. This presents with a broad spectrum of 55 complaints, such as fatigue, dyspnea, and even neuropsychiatric and gastrointestinal manifestations. Despite the variability of risk, the most worrisome feature of this sequence is that 80% of COVID‐19 patients experience them following the resolution of the primary infection [6]. This has also never been studied at large in the Palestinian population. Despite extensive research, the long‐term consequences of COVID‐19 remain unclear, especially in the Middle East.

As such, we conducted a cross‐sectional study on Palestinians residing in the West Bank. The study focused on individuals who tested positive for COVID‐19 between November and December 2020, with a randomized sample of 407 individuals out of a total of 8121 confirmed cases. Our study aims to shed light on the impact of COVID‐19 on this population and help healthcare professionals better understand the virus’s long‐term effects.

## 2. Methods

### 2.1. Study Design and Population

This is a cross‐sectional study that targeted COVID‐19 cases [confirmed by the Palestinian Ministry of Health (MOH)] that were infected from November 25th, 2020, to December 15th, 2020, in the West Bank (*n* = 8121). The determination of the sample size was guided by a desired margin of error of 5% and a confidence level of 95%. Given the West Bank population size of 3,200,000 and assuming a response distribution of 50%, the minimum recommended sample size is 385. A homogenous sample of 407 patients (age > 18) was selected from the COVID‐19 case registry to participate through randomized sampling. The study excluded patients who died prior to data collection, were under the age of 18, and refused to participate or failed to respond. In addition, patients who were difficult to talk to owing to psychotic disorders or dementia were all excluded. After the purpose of the study and the data collection procedures were all clarified to study participants, all respondents gave verbal informed consent on the phone to participate voluntarily and were given the freedom to abstain from answering or withdraw at any time during the study timeline. The study’s protocol was approved by the MOH and by the research ethics committee at Al‐Quds University (Ref. No. 183/REC/2021).

### 2.2. Data Collection

The data were collected in October of 2021. Each patient had an infection at least 10 months prior. Contact information for the participants, including phone number, gender, and place of residence, was obtained from the MOH electronic database. A questionnaire in Arabic was used to collect data through a phone call interview and was filled out by trained data collectors. The questionnaire was translated by a professional translator and reviewed by two clinical experts for accuracy. It included demographics, past medical history, questions about infection symptoms (duration, pattern, and severity), questions about infection consequences (the need for oxygen supplementation and hospital admission), and questions related to physical activity before and after the infection as well as reinfection. It was adapted from the Patient‐Led Research Collaborative with optimizations for the local population. Kindly find the questionnaire attached in the supporting information (available here) of this manuscript. The results of the aforementioned questionnaire were published in the study “Characterizing Long COVID in an International Cohort: 7 months of Symptoms and Their Impact” [7]. All the collected data were based on patients’ self‐reports, and no medical records were used. Data collectors had standardized training and adhered to a uniform interview process. They were blinded to the study hypotheses and participant outcomes to prevent bias. The assessment of inter‐rater reliability revealed a high level of agreement.

### 2.3. Statistical Analysis

Descriptive statistics were used to characterize the sample. We referred to the mean and standard deviation to characterize continuous variables, and frequencies and percentages for categorical variables. The duration of infection symptoms was recorded into three categories: no symptoms, ≤ 4 months, and > 4 months. A chi‐square test was used to identify the relationship between the persistence of infection symptoms and two variables: the need for hospitalization and recovery time. We also employed multivariate regression models to account for confounding variables in the prediction of these outcomes. Recovery time was defined as the self‐reported duration from the onset of the COVID‐19 infection until the individual subjectively perceived a return to their pre‐COVID level of daily activity and functioning. This measure reflects the participants’ overall sense of recovery and their return to pre‐illness baseline, rather than resolution of specific symptoms. SPSS version 25.0 was used for data analysis. The statistical significance was set at *p* < 0.05.

## 3. Results

### 3.1. Demographic and Clinical Characteristics of Study Participants

This study included 407 patients with a mean age of 40.0 (SD, 13.8). Out of these, 220 (54%) were female patients; 286 (70.3%) had no pre‐existing medical conditions; 45 (11.1%) had hypertension; 31 (7.6%) had Type II diabetes mellitus; and 13 (3.2%) had vitamin D deficiency. There were no cases of immunodeficiency or chronic kidney disease. Table 1 shows demographic data and significant clinical correlations.

**Table 1:** Demographic data and pre‐existing conditions.

**Demographic data**	

Age (year)	40.0 ± 13.8
Sex, females *n*, %	220, 54.1
BMI (kg/m^2^)	27 ± 11

**Pre-existing conditions**	

Asthma, *n*, %	9, 2.2
COPD, *n*, %	1, 0.2
Diabetes, *n*, %	31, 7.6
Cancer (any type), *n*, %	3, 0.7
Hypertension, *n*, %	45, 11.1
Rheumatoid arthritis, *n*, %	8, 2.0
Vitamin D deficiency, *n*, %	13, 3.2

### 3.2. Patients’ Symptoms and Their Need for Medical Services During COVID‐19 Infection

The results showed that 28.5% of the patients had chest pain; 34.6% had shortness of breath; 36.7% had cough; 36.4% had chills; 44.2% had fever; 51.8% had headache; 24.8% had diarrhea; 61.9% had anosmia; 9.6% had taste loss; 38.3% had appetite loss; 50.6% had lethargy; 64.9% had fatigue; 52.6% had joint pain; and 38.2% had muscle weakness. According to medical services, 7.6% needed hospitalization, 5.9% needed intubation or O_2_ therapy, and 14.0% had a chest x‐ray. Table 2 shows patients’ symptoms and their long‐term persistence.

**Table 2:** Patients’ COVID‐19 symptoms and its long‐term persistence.

Symptom	No (%)	Yes (%)	≤ 4 months (%)	> 4 months (%)
Chest pain	71.5	28.5	22.6	5.9
Shortness of breath	65.4	34.6	27.6	7
Cough	63.3	36.7	33.3	3.4
Chills/sweating	63.6	36.4	33.2	3.2
Intermittent fever	55.8	44.2	43.7	0.5
Headache	48.2	51.8	46.4	5.4
Diarrhea	75.2	24.8	23.9	0.9
Anosmia	38.1	61.9	55.7	6.2
Taste loss	90.4	9.6	4.8	4.8
Appetite loss	61.7	38.3	35.1	3.2
Lethargy	49.4	50.6	42.5	8.1
Fatigue	35.1	64.9	54.3	10.6
Joint pain	47.4	52.6	42	10.6
Muscle weakness	61.8	38.2	31	7.2

### 3.3. Patients’ Status After COVID‐19 Infection

92.6% of patients recovered within 4 months; 4.7% recovered within more than 4 months; and 2.7% had not recovered by the end of the study period. There was also a varying degree of recovery, with symptoms completely subsiding in 37.2% of patients, symptoms subsiding then recurring at various timestamps in 4.8% of patients, 40.3% of which had their symptoms fluctuate throughout this period, and ultimately 17.6% of the sample not having a change in symptoms before recovery. Regarding pre‐infection conditions, our sample reported an improvement of the conditions in 16.7% of cases, a decline in 13.3% of cases, and no change in 48% of cases. The remainder of these patients experienced variable improvements in certain conditions, a decline in others, and no change in the rest.

### 3.4. Correlation Between the Persistence of Symptoms and Hospitalization Need

Table 3 shows the results of the chi‐square test for checking the correlation between the persistence of COVID‐19 symptoms and hospitalization needs as a result of the infection. The duration of symptoms, which was defined as the amount of time that symptoms remained after the recovery from the acute COVID‐19 infection, was stratified into three categories (no symptoms, < 4 months, and > 4 months). Our results showed that the persistence of chest pain (*χ* ^2^, 16.225; *p* < 0.001), shortness of breath (*χ* ^2^, 13.257; *p* < 0.001), and lethargy (*χ* ^2^, 8.194; *p* = 0.017) was found to be significantly associated with hospitalization need. We developed a logistic regression model to correct for confounders in predicting hospitalization. The model had a null deviance of 219.22, a residual deviance of 187.83, and an AIC of 207.83. Male sex was the first associated factor to significantly increase the risk of admission (OR = 6.45, 95% CI: 2.16–20.5, *p* < 0.001). Height was another significant predictor, with each centimeter associated with a decreased odds of admission (OR = 0.94, 95% CI: 0.89–1.00, *p* = 0.039). The absence of shortness of breath was a significant protective factor, reducing the odds of admission by approximately 70% (OR = 0.29, 95% CI: 0.11–0.74, *p* < 0.001). Other variables were also important predictors, although they were not statistically significant, including chest pain and arthralgia. Although prior medical history was associated with an increased likelihood of admission, it was not significant.

**Table 3:** Correlation between the persistence of symptoms and hospitalization need.

Symptom		Hospitalization need (%)	Chi‐square (*χ* ^2^)	*p* value
Chest pain	No symptom	4.5	16.225	< 0.001
	≤ 4 months	13		
	> 4 months	25		

Shortness of breath	No symptom	4.1	13.257	**<** **0.001**
	≤ 4 months	13.4		
	> 4 months	17.5		

Cough	No symptom	5.9	0.59	0.274
	≤ 4 months	8.8		
	> 4 months	21.6		

Chills	No symptom	6.5	1.478	0.478
	≤ 4 months	8.8		
	> 4 months	15.4		

Intermittent Fever	No symptom	6.6	1.645	0.439
	≤ 4 months	8.4		
	> 4 months	49.1		

Headache	No symptom	7.7	0.663	0.718
	≤ 4 months	8		
	> 4 months	4.5		

Diarrhea	No symptom	6.5	2.667	0.264
	≤ 4 months	10.2		
	> 4 months	27.3		

Anosmia	No symptom	9	0.797	0.671
	≤ 4 months	6.6		
	> 4 months	7.9		

Taste loss	No symptom	4.1	0.464	0.793
	≤ 4 months	71.7		
	> 4 months	10.2		

Appetite loss	No symptom	6.8	2.703	0.259
	≤ 4 months	9.8		
	> 4 months	0		

Lethargy	No symptom	4	8.194	**0.017**
	≤ 4 months	9.8		
	> 4 months	18.2		

Fatigue	No symptom	7.7	1.208	0.547
	≤ 4 months	6.3		
	> 4 months	13.9		

Joint pain	No symptom	4.7	5.999	0.05
	≤ 4 months	8.8		
	> 4 months	16.2		

Muscle weakness	No symptom	6.4	3.937	0.14
	≤ 4 months	11.1		
	> 4 months	3.4		

*Note:* Fisher’s exact test was used where appropriate. Bold values indicate statistically significant values.

### 3.5. Correlation Between the Persistence of Symptoms and Recovery Time

Table 4 shows the findings of the chi‐square test for checking the correlation between the persistence of COVID‐19 symptoms and recovery time from the infection. The duration of symptoms and recovery were stratified into three categories (no symptoms, < 4 months, and > 4 months). According to the *p* value seen in Table 4, at the 0.05 level of confidence, our results showed that the persistence of all the mentioned symptoms was found to be significantly associated with the duration of recovery. We fitted a multinomial logistic regression model to predict recovery time. The model included anosmia, headache, and cough, with an AIC of 218.67 and a residual deviance of 186.67. Anosmia lasting longer than 4 months was significantly associated with longer recovery times (*p* = 0.006), with headaches lasting for more than 4 months carrying a similar effect (*p* = 0.003). Cough lasting for longer than 4 months was borderline significant with a similar effect (*p* = 0.067). Past medical history was not significantly correlated (*p* = 0.420).

**Table 4:** Correlation between the persistence of symptoms and recovery time.

Symptom		Recovery time (%)			Chi‐square (*χ* ^2^)	*p* value
		Did not return	≤ 4 months	> 4 months		
Chest pain	No symptom	1.7	94.8	3.4	20.754	< 0.001
	≤ 4 months	3.4	92	4.5		
	> 4 months	10.7	71.4	17.9		

Shortness of breath	No symptom	1.5	95.9	2.6	29.023	**<** **0.001**
	≤ 4 months	1.9	91.3	6.7		
	> 4 months	13.5	73	13.5		

Cough	No symptom	1.6	96.9	1.6	58.667	**<** **0.001**
	≤ 4 months	1.6	89.9	8.6		
	> 4 months	23.8	57.1	19		
Chills	No symptom	1.5	94.6	3.9	19.694	**<** **0.001**
	≤ 4 months	3	91	6		
	> 4 months	20	73.3	6.7		

Intermittent Fever	No symptom	1.3	95.2	3.5	10.48	**0.033**
	≤ 4 months	4	89.7	6.3		
	> 4 months	20	80	0		

Headache	No symptom	0.5	98	1.5	56.383	**<** **0.001**
	≤ 4 months	2.2	91.3	6.5		
	> 4 months	22.2	63	14.8		

Diarrhea	No symptom	1.6	96.1	2.3	46.215	**<** **0.001**
	≤ 4 months	4.2	83.3	12.5		
	> 4 months	40	60	0		

Anosmia	No symptom	2.6	96.1	1.3	11.718	**0.02**
	≤ 4 months	1.9	91.7	6.5		
	> 4 months	8.3	83.3	8.3		

Taste loss	No symptom	2.9	95.4	1.7	10.435	**0.034**
	≤ 4 months	1.9	91.8	6.3		
	> 4 months	7.7	80.8	11.5		

Appetite loss	No symptom	2.8	94.8	2.4	14.433	**0.006**
	≤ 4 months	1.4	90	8.6		
	> 4 months	12.5	81.3	6.3		

Lethargy	No symptom	1.5	96	2.5	31.686	**<** **0.001**
	≤ 4 months	2.4	93.9	3.7		
	> 4 months	9.5	71.4	19		

Fatigue	No symptom	0.7	99.3	0	38.17	**<** **0.001**
	≤ 4 months	1.9	92.9	5.2		
	> 4 months	11.1	74	14.8		

Joint pain	No symptom	1	97.4	1.6	37.053	**<** **0.001**
	≤ 4 months	1.2	91.5	7.3		
	> 4 months	14.3	77.6	8.2		

Muscle weakness	No symptom	2	96	2	24.977	**<** **0.001**
	≤ 4 months	1.6	90.2	8.1		
	> 4 months	12.5	75	12.5		

*Note:* Fisher’s exact test was used where applicable. Bolded values are statistically significant.

## 4. Discussion

Our study aimed to investigate the duration and severity of COVID‐19 symptoms among individuals in the West Bank following resolution of their acute infection. The results offer insights into the demographic and clinical characteristics of participants, symptom patterns, healthcare utilization during acute illness, and correlations between persistent symptoms, hospitalization, and recovery time.

Our questionnaire included thirty long‐term symptoms covering respiratory, cardiovascular, gastrointestinal, musculoskeletal, neurological, psychological, and other general and miscellaneous symptoms assessed from 1 week up to 10 months after recovery.

While many participants had no prior chronic illnesses, hypertension, Type II diabetes mellitus, and vitamin D deficiency were observed among the patients. Previous studies have linked these comorbidities to an elevated risk of persistent long COVID‐19 symptoms [8]. These findings highlight the necessity of accounting for demographic and clinical characteristics when evaluating the duration and severity of COVID‐19 sequelae.

A wide spectrum of symptoms was reported by participants both during and after COVID‐19 infection, with fatigue, anosmia, joint pain, and headache among the most frequently cited. Fatigue, in particular, was the most prevalent and persistent symptom in our cohort, affecting 64.9% of participants. This finding aligns with the results of a large meta‐analysis by Lopez‐Leon et al., which identified fatigue as the most common long‐term effect of COVID‐19, with a prevalence of 58% among survivors [6]. Similarly, Khodeir et al. reported a prevalence of 73% in their cohort [9]. In our study, fatigue persisted for more than 4 months in 10.6% of patients.

Fatigue is recognized as a complex and debilitating symptom not only in long COVID but also in various neurological and oncological disorders. Its pathogenesis is multifactorial and cannot be attributed to a single underlying mechanism [10]. Proposed contributors include alterations in neurotransmitter levels, chronic inflammation, psychiatric comorbidities, psychosocial burden, cognitive dysfunction, and disturbances in the substrate metabolism and availability. Recent consensus defines post‐COVID‐19 fatigue as a reduction in physical and/or mental performance resulting from central, psychological, and/or peripheral factors attributable to COVID‐19 [11]. The high prevalence and persistence of fatigue observed in our cohort underscore its role as a hallmark of long COVID, with substantial implications for quality of life and the need for targeted rehabilitation strategies.

Lethargy refers to a state of sluggishness, inactivity, and apathy that encompasses reduced mental and physical alertness [12]. Although this definition distinguishes lethargy from fatigue, the literature often conflates these symptoms or assesses them jointly, which can hinder precise epidemiological interpretation. In a large‐scale global web‐based survey, fatigue and lethargy were reported as a single composite symptom, with 59% of respondents experiencing one or both [13]. By contrast, in a prospective cohort study, lethargy was evaluated as a distinct symptom and was identified as an independent risk factor for the development of long COVID [14]. In our study, lethargy was reported by 50.6% of participants, with persistence beyond 4 months in 10.6%. Collectively, these findings emphasize the need for future research to employ consistent and precise symptom definitions to better delineate the unique contributions and clinical significance of lethargy in long COVID.

The second most prevalent symptom in our study was anosmia. The mechanism of anosmia caused by viral infection is unknown, but it is believed that the virus damages the olfactory epithelium or causes an inflammatory reaction that prevents the neuronal regeneration of the olfactory epithelium [15]. It has been noted in human coronavirus infections in the past, but SARS‐CoV‐2 infection is more likely to cause it [9]. In our study, 61.9% of the people questioned suffered from anosmia. Of these, 55.7% persisted for less than 4 months, while 6.2% experienced a longer decline. Although dysgeusia, or loss of taste, is frequently present with anosmia, it is now regarded as a cardinal and significant symptom of COVID‐19 [9]. Our study indicated that unexpectedly, only 9.6% of our sample had dysgeusia. This may be attributed to the presence of different mutations of COVID‐19, in addition to the differing pathogenicity of SARS‐COV‐2.

Joint pain was also commonly reported, affecting 52.6% of respondents, with persistence beyond 4 months in 10.6% of cases. This finding is consistent with prior systematic reviews indicating that post‐COVID‐19 arthralgia may persist from 4 weeks up to 12 months, with prevalence estimates ranging from 2% to 65% [16]. Post‐COVID‐19 arthralgia was treated with nonsteroidal anti‐inflammatory medications and local steroids, indicating a possible nociceptive phenotype because of the activation of peripheral nociceptors. Furthermore, it was hypothesized that SARS‐CoV‐2‐induced injury to connective tissue might result in diffuse pain, exhibiting nociplastic characteristics, particularly in individuals with hypermobile joints [16].

Neuropsychiatric manifestations associated with COVID‐19 are multifactorial and complicated in etiology. The inflammatory reaction to the infection, cerebrovascular disease (including hypercoagulation), the hypoxic state, drug side effects, and the psychosocial elements of being ill with a stigmatized infection might all be connected to the etiology [6]. One prominent early neurological sign of COVID‐19 across all epidemiological investigations is headache [17, 18]. Headache was reported in 51.8% of patients in our study. In other studies, the percentages ranged between 44% and 64% [6, 8]. Hospitalization did not correlate significantly with the persistence of headache. However, it was significantly associated with recovery time.

The available research suggests that there are two possible explanations for the pathophysiology of headache as a chronic symptom of COVID‐19. Hypoxia and/or hypercapnia were initially taken into consideration while examining the etiology of COVID‐19 headaches [19, 20]. This theory was tested in a group of seventy patients who were monitored for 3 months following the acute stage. However, they concluded that hypoxia was most likely not implicated in long COVID headache [21]. As for the second theory, individuals who experience prolonged COVID‐19 headaches may exhibit a biohumoral reaction and chronic immune system activation, as seen by changes in cytokine and interleukin levels in the blood [20]. One exploratory study evaluating the correlation between cytokine profiles and headache status in COVID‐19, IL‐10, IL23, and PIGF1 was ascertained in the pathophysiological process [22].

The clinical and public health implications of persistent headache in post‐COVID‐19 are considerable, particularly in resource‐constrained settings. Chronic headache can negatively impact functional capacity, work productivity, and quality of life, while also increasing demand for outpatient and specialty care. As such, systematic neurological assessment and tailored multidisciplinary management of persistent headache should be prioritized in long COVID care pathways. These strategies are especially vital in settings with limited access to specialized care, where untreated or undertreated neuropsychiatric sequelae can impose a significant burden on individuals and health systems alike.

Psychosocial symptoms associated with long COVID, including sleep disturbances, anxiety, depression, and impaired concentration, remain incompletely characterized and often underappreciated in both research and clinical care. Studies have reported a wide prevalence of sleep disturbances, with rates from 34% to 50% and some data indicating rates as high as 41.3% with a strong association with hospitalization [23]. In contrast, our sample demonstrated a lower prevalence of sleep disturbances (27.5%) and found no significant correlation with hospitalization. Similarly, the occurrence of depressive symptoms in our cohort closely aligns with findings from recent meta‐analyses, which have questioned the magnitude of the association between long COVID and depressive symptoms [24]. These observations suggest that psychological sequelae may be influenced by a complex interplay of factors, including the acute psychosocial impact of illness, societal stigma, and individual coping mechanisms. Our finding that early clinical recovery was associated with the resolution of psychosocial symptoms highlights the value of timely supportive care and psychosocial interventions. In resource‐limited contexts, integrating mental health assessment and support into long COVID care is critical to addressing the full spectrum of long‐term sequelae and reducing the broader societal and economic burden of the pandemic.

Following the resolution of the infection, a substantial impact on the gastrointestinal system is observed. It is unclear what causes the gastrointestinal signs and symptoms. Still, some studies discuss the virus’s cytotoxic effect, cytokine release, local or systemic inflammation, or changes in the intestinal microbiota [25]. Parasa et al. reported that SARS‐CoV‐2 RNA‐positive stool was detected in 40.5% of their sample even after recovering from respiratory symptoms [26]. A similar meta‐analysis found that 12% of patients reported digestive disorders, implying a significant link between COVID‐19 and long‐term gastrointestinal consequences [6]. In our study, gastrointestinal symptoms were slightly more prevalent than previously reported. For example, diarrhea occurred at a rate of 24.8%, with the majority lasting at least 4 months. Other symptoms included abdominal pain in 15.5% and loss of appetite in 35.1%. A study conducted by Zaho et al. in China discovered that the overall incidence of gastrointestinal symptoms was 18.6%, with diarrhea accounting for 13.5% and abdominal pain accounting for only 5.7% [27]. We can attribute the discrepancy between our findings and the literature to a variety of confounders, including demographic characteristics, eating habits, food types in Middle Eastern countries, and genetic predisposition of Middle Eastern people. Moreover, the difference between COVID‐19 strains also has a role, as some strains had a significant impact on certain systems, causing some signs and symptoms or consequences to appear more frequently than others.

Chest pain is the leading symptom of pulmonary and cardiac diseases [28]. In our study, the prevalence of chest pain during the acute phase of the infection was 28.5%. Out of these patients, chest pain persisted for less than 4 months in 22.6%, and for more than 4 months in 5.9%. This is congruent with the results of a study by Huang et al., which demonstrated that chest pain persisted in 7% of patients 1 year after their initial infection with COVID‐19 [29]. In a review of 69 studies by Elhiny et al., about COVID‐19 complications after recovery, cardiovascular symptoms such as chest pain and palpitations were one of the most common complications [30]. The need for hospitalization correlated significantly with chest pain in our study. The persistence of chest pain after the initial acute infection could be attributed to the fact that the SARS‐CoV‐2 virus leads to an increase in the myocardial demand, in addition to causing ongoing inflammation within the heart [31, 32]. Dani et al. hypothesized that long‐term cardiac symptoms, such as chest pain, are caused by the autonomic nervous system instability as a result of viral destruction or immunological response [33, 34].

In a study on the long‐term effects of COVID‐19 on cardiac symptoms, dyspnea (shortness of breath) was identified as a significant cardiovascular symptom, reported in 41.6% of patients even after one year of infection [34]. This aligns with the results of our study, where dyspnea is a prevalent symptom affecting 34.6% of patients. Of these, 27.6% experienced dyspnea for less than 4 months and 7% for more than 4 months. The need for hospitalization due to dyspnea was notably significant in our study. Dyspnea often persists long after a COVID‐19 infection due to cardiopulmonary complications, inflammatory responses, vascular damage, muscle deconditioning, and psychological factors. Women and individuals with pre‐existing conditions such as coronary artery disease are more likely to experience severe and persistent dyspnea. Given its impact on daily functioning and recovery, systematic assessment and individualized management of dyspnea should be integrated into follow‐up care for COVID‐19 survivors [35].

In addition to evaluating the long‐term symptoms of COVID‐19, our study also evaluated the need for medical services among the patients. Hospitalization was required for 7.6% of the participants, indicating the severity of the disease in a subset of individuals. Additionally, 5.9% of patients needed interventions such as intubation or oxygen therapy and 14% had chest x‐rays. These findings underscore the importance of healthcare resources and support for individuals with severe COVID‐19 symptoms.

The status of patients after recovery from COVID‐19 was also assessed in the study. The majority of patients (92.6%) achieved recovery within 4 months, while a small percentage (2.7%) did not recover during the study period. Among those who recovered, a substantial proportion experienced complete subsidence of symptoms, while a smaller percentage reported fluctuating symptoms or persistent symptoms of the same intensity and frequency. Importantly, the study revealed that pre‐existing conditions influenced the outcomes, with some individuals experiencing worsening of their conditions, while others showed improvement or remained stable.

A large multicenter cohort study demonstrates that fluctuating pain symptoms are a common feature of long COVID, imposing a persistent burden on patients’ quality of life and creating uncertainty regarding the recovery process. Among previously hospitalized COVID‐19 survivors, the prevalence of musculoskeletal pain declined over 18 months, but a substantial proportion of patients experienced recurrent or newly developing pain after initial recovery, reflecting a nonlinear and unpredictable trajectory. These symptom fluctuations were more frequent in women, individuals with pre‐existing pain conditions, and those with longer hospitalizations. The evolving nature of post‐COVID pain underscores the importance of longitudinal monitoring and flexible, individualized management approaches to meet the needs of affected patients [36].

The results demonstrated that the persistence of chest pain, shortness of breath, and lethargy was significantly associated with the need for hospitalization. This finding highlights the importance of monitoring and addressing these symptoms promptly to prevent severe disease progression.

Beyond clinical outcomes, persistent COVID‐19 symptoms exert a substantial economic and social toll. Studies estimate that long COVID may result in significant productivity losses and increased healthcare utilization, with downstream impacts on health systems and household income [37]. These burdens are particularly pronounced in resource‐constrained settings, where limited infrastructure and reduced access to comprehensive care further exacerbate the impact of long COVID. The psychosocial burden, especially among those with inadequate access to support services, underscores the urgent need for integrated policy approaches that address both medical and socioeconomic recovery [38, 39].

## 5. Limitations

The study has certain limitations that provide important considerations for interpreting the findings and offer directions for future research. First, data collection relied exclusively on phone consultations without accompanying clinical, radiological, or laboratory validations. This method could introduce misclassification bias, particularly for subjective symptoms such as fatigue, lethargy, or headache, thereby impacting the accuracy of the reported findings.

Reliance on self‐reported symptom data also introduces potential recall bias, as participants may not accurately recall or report the duration and severity of symptoms especially after at least 10 months after the initial infection. Additionally, the study’s sample selection process may introduce selection bias, as participants may be more likely to have ongoing or more severe symptoms, leading to an overestimation of symptom prevalence. Furthermore, the absence of longitudinal follow‐up precludes the assessment of symptom progression or resolution over time.

Moreover, some patients with negative PCR test results but typical clinical and radiological signs of COVID‐19 may have been missed in the dataset. This could lead to an underrepresentation of certain individuals who experienced COVID‐19 symptoms.

Another limitation is the study design. As a cross‐sectional investigation conducted after resolution of the primary COVID‐19 infection, it captures data at a single time point, which precludes the ability to establish temporal relationships or infer causality. Although the study identified statistically significant associations, such as between symptom persistence and hospitalization, these should not be interpreted as evidence of causal relationships but rather as preliminary findings that warrant further longitudinal investigation.

The study was unable to stratify results by the SARS‐CoV‐2 variant type, as genomic sequencing was not performed. Given that data collection occurred during a period of emerging and cocirculating variants, this may have introduced variability in symptom patterns and illness severity. Psychosocial factors were also not assessed, which may have led to an underestimation of their influence on symptom manifestation and persistence.

Finally, as the study primarily included individuals with moderate to severe illness, the generalizability of these findings to asymptomatic or mildly affected populations remains limited.

## 6. Conclusion

In conclusion, this study provides important insights into the long‐term symptoms experienced by individuals recovering from COVID‐19. It also stratifies the demographics and clinical characteristics of the study sample. In addition, it provides insights into the symptoms experienced by the participants during their infection, their medical service requirements, their health status after recovering from COVID‐19, and the relationships between the persistence of symptoms, the need for hospitalization, and the duration of recovery.

These results have implications for healthcare professionals, policymakers, and individuals affected by COVID‐19, emphasizing the need for comprehensive care plans, resource allocation, and ongoing support. We believe future research should focus on further characterization of the delayed symptoms seen postresolution of COVID‐19 infection, while also tackling the limitations of previous studies, to further elaborate potential mechanisms and interventions to improve the management and outcomes for COVID‐19 survivors.

## Ethics Statement

This project was ethically approved by the Al‐Quds University Ethics Committee under Ref. No. 183/REC/2021.

Consent for participation in the study and the publication of the manuscript was taken from the patients themselves prior to data collection.

## Conflicts of Interest

The authors declare no conflicts of interest.

## Author Contributions

Tareq Jarrar: data curation, investigation, validation, visualization, and writing–original draft; Noor Halaseh: data curation, investigation, writing–original draft, and writing–review and editing; Duha Doudin: data curation, investigation, writing–original draft, and writing–review and editing; Peter Bael: investigation, validation, visualization, formal analysis, writing–original draft, and writing–review and editing; Ahmad Abu Sada: conceptualization, data curation, investigation, methodology, and funding acquisition; Atheer Shaheen: data curation, investigation, methodology, and writing–original draft; Eyad Jobeh: data curation, investigation, methodology, and writing–original draft; Ahmad Abu Awwad: data curation, investigation, methodology, and funding acquisition; Bayan Alqtishat: investigation, methodology, validation, visualization, formal analysis, and writing–review and editing; and Hussein Hallak: conceptualization, investigation, methodology, validation, visualization, formal analysis, and writing–review and editing.

## Funding

No funding was received for this research.

## Supporting information


**Supporting Information** Additional supporting information can be found online in the Supporting Information section.

## Data Availability

The data are available upon request from the corresponding author.
