# Supplementary material for: Long‐Term Self‐Reported Symptoms Among Adults After COVID‐19 Infection in the West Bank: A Cross‐Sectional Analysis
Source: Glob Health Epidemiol Genom. 2025 Dec 11;2025:2867843. doi: 10.1155/ghe3/2867843 (PMC12782249; doi:10.1155/ghe3/2867843)
Supplement: Supplementary file 1 — Supporting Information Additional supporting information can be found online in the Supporting Information section. [file GHE3-2025-2867843-s001.docx]

## **Long-Term Self-Reported Symptoms Among Adults After COVID-19 Infection in the West Bank: A Cross-Sectional Analysis**

## **Participant Information and Consent**

We are researchers from Al-Quds University conducting a study on post-COVID-19 complications in the West Bank.

 Please note:
 - Participants must be 18 years or older.
 - All information will be confidential and used only for research purposes.
 - Participation is voluntary, with no compensation.
 - You may withdraw at any time without penalty.
 - Estimated completion time: 5-20 minutes.

## **Consent Confirmation**

QNo:

A. Phone number: _________________

B. I was infected with Coronavirus (COVID-19) and I agree to participate in this questionnaire after reading the introduction.

· 1. Yes

· 2. No

## **General Information**

Sex:

· 1. Male

· 2. Female

Date of birth: ___________ (to be scaled)

Height (cm): ____________

Weight (kg): ____________ (to be converted to BMI)

## **Pre-Existing Conditions**

Check the pre-existing conditions that you had when you tested positive for COVID-19 (Check all that apply):

· G01 - I didn't have any pre-existing condition

· G02 - Asthma

· G03 - COPD

· G04 - Diabetes

· G05 - Cancer (any type)

· G06 - High blood pressure

· G07 - Pre-existing Auto-immune disease

· G08 - Vitamin D deficiency

· G09 - Anemia

· G10 - Other: __________

## **Physical Activity Before Infection**

How physically active were you prior to the onset of symptoms?

· 1. Very physically active

· 2. Moderately physically active (do my daily tasks without physical fatigue)

· 3. Unstable physical condition (physical condition fluctuates)

· 4. Mostly sedentary

· 5. Sedentary

## **Hospitalization and Treatment During Infection**

Did you go to the hospital due to symptoms of Coronavirus?

· 1. Yes

· 2. No

If yes, how long did you stay in the hospital (days)? _________

Were you put on a ventilator or O2 therapy during the acute phase?

· 1. Yes

· 2. No

Were you asked to get a chest X-ray during the acute phase?

· 1. Yes

· 2. No

## **Symptoms Persisting After Recovery**

What symptoms started during your acute illness and persisted after recovery (for more than one week)?

· M01 - No symptoms persisted beyond the acute phase

· M02 - General weakness

· M03 - Fever

· M04 - Unwillingness or inability to walk

· M05 - Muscle pain

· M06 - Loss of taste/smell(Anosmia)

· M07 - Sleep difficulties

· M08 - Shortness of breath

· M09 - Headache

· M10 - Diarrhea

· M11 - Other: __________

## **Change in Pre-Existing Conditions**

Did any of your pre-existing conditions change during the course of your COVID-19 symptoms?

· 1. Yes, they got worse.

· 2. Yes, they got better.

· 3. Some got better, some stayed the same, some got worse.

· 4. No, they stayed the same.

· 5. N/A (I did not have any pre-existing condition)

## **Symptom Timeline After Recovery**

Mark the duration of symptoms (if applicable) for each of the following periods:

|  | N\A (I did not have this symptom) | Week 1 and week 2 | Week 3 and 4 | Month 2 | Month 3 and month 4 | Month 5 and month 6 | Month 7 and month 8 | Month 9 and month 10 | After more than 10 months |
| --- | --- | --- | --- | --- | --- | --- | --- | --- | --- |
| Chest pain |  |  |  |  |  |  |  |  |  |
| Shortness of breath |  |  |  |  |  |  |  |  |  |
| Cough |  |  |  |  |  |  |  |  |  |
| Chills/ sweating |  |  |  |  |  |  |  |  |  |
| Fever |  |  |  |  |  |  |  |  |  |
| Headache |  |  |  |  |  |  |  |  |  |
| Diarrhea |  |  |  |  |  |  |  |  |  |
| Anosmia |  |  |  |  |  |  |  |  |  |
| Taste loss |  |  |  |  |  |  |  |  |  |
| Appetite loss |  |  |  |  |  |  |  |  |  |
| Lethargy |  |  |  |  |  |  |  |  |  |
| Fatigue |  |  |  |  |  |  |  |  |  |
| Joint pain |  |  |  |  |  |  |  |  |  |
| Muscle weakness |  |  |  |  |  |  |  |  |  |

## **Nature of Symptoms During Recovery**

Describe the nature of your symptoms during the recovery period (Check all that apply):

· P01 - Symptoms fluctuated in intensity and frequency. (Yes=1, No=0)

· P02 - New symptoms appeared at different times. (Yes=1, No=0)

· P03 - I had the same symptoms the entire time. (Yes=1, No=0)

· P04 - All symptoms subsided completely (for 1 week or longer). (Yes=1, No=0)

· P05 - I did not have any symptoms. (Yes=1, No=0)

· P06 - Other: ___________________________

## **Post-Recovery Status**

How physically active are you after recovery?

· 1. Very physically active

· 2. Moderately physically active (do my daily tasks without physical fatigue)

· 3. Unstable physical condition (physical condition fluctuates)

· 4. Mostly sedentary

· 5. Sedentary

Did you use O2 therapy after recovery?

· 1. Yes

· 2. No

If you returned to your normal life and activity, how long did it take (recovery time)?

· 1. I did not return to my normal life.

· 2. 1 to 2 weeks.

· 3. 2 to 4 weeks.

· 4. 1 to 2 months.

· 5. 3 to 4 months.

· 6. 5 to 6 months.

· 7. 7 to 8 months.

· 8. 9 to 10 months.

· 9. More than 10 months.

Have you tested PCR-positive for COVID-19 after recovery?

· 1. Yes

· 2. No

## **Long-Term Self-Reported Symptoms Among Adults After COVID-19 Infection in the West Bank: A Cross-Sectional Analysis**

## **Participant Information and Consent**

We are researchers from Al-Quds University conducting a study on post-COVID-19 complications in the West Bank.

 Please note:
 - Participants must be 18 years or older.
 - All information will be confidential and used only for research purposes.
 - Participation is voluntary, with no compensation.
 - You may withdraw at any time without penalty.
 - Estimated completion time: 5-20 minutes.

## **Consent Confirmation**

QNo:

A. Phone number: _________________

B. I was infected with Coronavirus (COVID-19) and I agree to participate in this questionnaire after reading the introduction.

· 1. Yes

· 2. No

## **General Information**

Sex:

· 1. Male

· 2. Female

Date of birth: ___________ (to be scaled)

Height (cm): ____________

Weight (kg): ____________ (to be converted to BMI)

## **Pre-Existing Conditions**

Check the pre-existing conditions that you had when you tested positive for COVID-19 (Check all that apply):

· G01 - I didn't have any pre-existing condition

· G02 - Asthma

· G03 - COPD

· G04 - Diabetes

· G05 - Cancer (any type)

· G06 - High blood pressure

· G07 - Pre-existing Auto-immune disease

· G08 - Vitamin D deficiency

· G09 - Anemia

· G10 - Other: __________

## **Physical Activity Before Infection**

How physically active were you prior to the onset of symptoms?

· 1. Very physically active

· 2. Moderately physically active (do my daily tasks without physical fatigue)

· 3. Unstable physical condition (physical condition fluctuates)

· 4. Mostly sedentary

· 5. Sedentary

## **Hospitalization and Treatment During Infection**

Did you go to the hospital due to symptoms of Coronavirus?

· 1. Yes

· 2. No

If yes, how long did you stay in the hospital (days)? _________

Were you put on a ventilator or O2 therapy during the acute phase?

· 1. Yes

· 2. No

Were you asked to get a chest X-ray during the acute phase?

· 1. Yes

· 2. No

## **Symptoms Persisting After Recovery**

What symptoms started during your acute illness and persisted after recovery (for more than one week)?

· M01 - No symptoms persisted beyond the acute phase

· M02 - General weakness

· M03 - Fever

· M04 - Unwillingness or inability to walk

· M05 - Muscle pain

· M06 - Loss of taste/smell(Anosmia)

· M07 - Sleep difficulties

· M08 - Shortness of breath

· M09 - Headache

· M10 - Diarrhea

· M11 - Other: __________

## **Change in Pre-Existing Conditions**

Did any of your pre-existing conditions change during the course of your COVID-19 symptoms?

· 1. Yes, they got worse.

· 2. Yes, they got better.

· 3. Some got better, some stayed the same, some got worse.

· 4. No, they stayed the same.

· 5. N/A (I did not have any pre-existing condition)

## **Symptom Timeline After Recovery**

Mark the duration of symptoms (if applicable) for each of the following periods:

|  | N\A (I did not have this symptom) | Week 1 and week 2 | Week 3 and 4 | Month 2 | Month 3 and month 4 | Month 5 and month 6 | Month 7 and month 8 | Month 9 and month 10 | After more than 10 months |
| --- | --- | --- | --- | --- | --- | --- | --- | --- | --- |
| Chest pain |  |  |  |  |  |  |  |  |  |
| Shortness of breath |  |  |  |  |  |  |  |  |  |
| Cough |  |  |  |  |  |  |  |  |  |
| Chills/ sweating |  |  |  |  |  |  |  |  |  |
| Fever |  |  |  |  |  |  |  |  |  |
| Headache |  |  |  |  |  |  |  |  |  |
| Diarrhea |  |  |  |  |  |  |  |  |  |
| Anosmia |  |  |  |  |  |  |  |  |  |
| Taste loss |  |  |  |  |  |  |  |  |  |
| Appetite loss |  |  |  |  |  |  |  |  |  |
| Lethargy |  |  |  |  |  |  |  |  |  |
| Fatigue |  |  |  |  |  |  |  |  |  |
| Joint pain |  |  |  |  |  |  |  |  |  |
| Muscle weakness |  |  |  |  |  |  |  |  |  |

## **Nature of Symptoms During Recovery**

Describe the nature of your symptoms during the recovery period (Check all that apply):

· P01 - Symptoms fluctuated in intensity and frequency. (Yes=1, No=0)

· P02 - New symptoms appeared at different times. (Yes=1, No=0)

· P03 - I had the same symptoms the entire time. (Yes=1, No=0)

· P04 - All symptoms subsided completely (for 1 week or longer). (Yes=1, No=0)

· P05 - I did not have any symptoms. (Yes=1, No=0)

· P06 - Other: ___________________________

## **Post-Recovery Status**

How physically active are you after recovery?

· 1. Very physically active

· 2. Moderately physically active (do my daily tasks without physical fatigue)

· 3. Unstable physical condition (physical condition fluctuates)

· 4. Mostly sedentary

· 5. Sedentary

Did you use O2 therapy after recovery?

· 1. Yes

· 2. No

If you returned to your normal life and activity, how long did it take (recovery time)?

· 1. I did not return to my normal life.

· 2. 1 to 2 weeks.

· 3. 2 to 4 weeks.

· 4. 1 to 2 months.

· 5. 3 to 4 months.

· 6. 5 to 6 months.

· 7. 7 to 8 months.

· 8. 9 to 10 months.

· 9. More than 10 months.

Have you tested PCR-positive for COVID-19 after recovery?

· 1. Yes

· 2. No
